# Participation of medical students in the medical team of sports events

**DOI:** 10.1002/jgf2.645

**Published:** 2023-08-22

**Authors:** Ayano Hamai, Tadao Okada, Kosuke Uemura, Takuro Uchida, Keita Kondo, Yuto Yamada

**Affiliations:** ^1^ Department of General Medicine Awa Regional Medical Center Chiba Japan; ^2^ Tessyokai Kameda Family Clinic Tateyama Chiba Japan; ^3^ Department of General Medicine Juntendo University Faculty of Medicine Tokyo Japan

## Abstract

Including medical student volunteers in the medical team for the triathlon strengthens the medical team structure, and encourages medical students to involve in sports medicine. Family physicians who play a role in the medical team for sports events can adopt medical students as a member and can educate them.

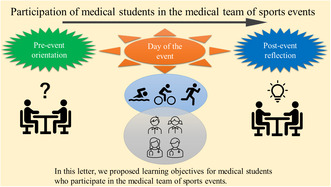

To the Editor,

Although sufficient staff and a first‐aid system are required to conduct safe sports events, they are usually unavailable. Family physicians can play a role in sports events;^1^ however, they usually require a large workforce. In Japan, emergency medical technician (EMT) students have contributed as part of a first‐aid team at a marathon event.^2^ A first‐response team by collegiate students was reported in the United States,^3^ but not in Japan, not to mention medical students. Medical students rarely have opportunities to experience sports medicine. Our city holds a triathlon competition annually, and we organize the medical team. Therefore, we aimed to include medical students in our medical teams.

The Tateyama‐Wakashio triathlon competition was held in May 2022, and 822 non‐elite athletes participated. In addition to 23 medical staff, we recruited six medical student volunteers for the medical team of the triathlon competition. Prior to the competition, we held an online orientation session for all the medical staff and students. We discussed first‐aid, injuries and illnesses commonly encountered in triathlons, and ways to handle them. There were five first‐aid stations, each comprising three to seven medical staff members and one or two students. In the competition, we treated 64 athletes (47 with injuries and 18 with illnesses). Medical students guided athletes, noted medical records, and assisted the medical staff. None of the athletes were seriously injured, and only one athlete had a wound that required suturing and was admitted to a hospital. After the event, a reflection session was held for students with medical staff to deepen their understanding of first aid and sports medicine. We discussed regarding sports and the necessary preparations for first aid.

After the process, we received feedback from the students and medical staff. Medical staff believed that having more members in a first‐aid station could help in several situations and contribute to safety and quality of care. A pre‐event medical simulation could allow students to assume more roles. All medical students were satisfied with their experiences. Some commented that they should be skilled in basic life support prior to the event.

Medical education scarcely covers sports medicine.^4^ The model core curriculum for medical education, which was revised in 2022, included a sports medicine curriculum for the first time in Japan. Participation in onsite sports events can serve as an early exposure program. It provides medical students with a highly satisfactory experience through appropriate guidance and feedback. An enjoyable training experience influences future career choices.^5^ However, while medical students have the potential to be part of medical teams, they are less skilled than EMT students. Therefore, a discussion regarding preparation and learning objectives is required. Table 1 shows the objectives that we propose for the medical students.

**TABLE 1:** Learning objectives expected for medical students who participate in the medical teams of sports events.

Timing	Detail
Pre‐event	By the end of the pre‐event orientation session, students will be able to: ‐ list the characteristics of the sport, rules, common injuries, and illnesses. ‐ recognize the role of the first‐aid team. ‐ explain how to respond to medical emergencies. ‐ explain how to treat common injuries and illnesses.
Day of the event	By the end of the day of the event, students will be able to: ‐ play a role in the first‐aid team with medical professionals. ‐ communicate with athletes, medical staff, and non‐medical event staff.
Post‐event	By the end of the reflection session, students will be able to: ‐ reflect on the activities of the medical team during the sports event. ‐ review responses to injuries and illnesses. ‐ discuss the role of the medical team at sports events.

By including medical student volunteers in the medical team for the triathlon, the medical team structure was strengthened, and the medical students gained a lot of knowledge. Family physicians who play a role in the medical team for sports events can adopt medical students as a member and can educate them.

## CONFLICT OF INTEREST STATEMENT

The authors declare no conflict of interest for this article.
